# Fundus image classification using Inception V3 and ResNet-50 for the early diagnostics of fundus diseases

**DOI:** 10.3389/fphys.2023.1126780

**Published:** 2023-02-15

**Authors:** Yuhang Pan, Junru Liu, Yuting Cai, Xuemei Yang, Zhucheng Zhang, Hong Long, Ketong Zhao, Xia Yu, Cui Zeng, Jueni Duan, Ping Xiao, Jingbo Li, Feiyue Cai, Xiaoyun Yang, Zhen Tan

**Affiliations:** ^1^ Health Management Center, Shenzhen University General Hospital, Shenzhen University Clinical Medical Academy, Shenzhen University, Shenzhen, Guangdong, China; ^2^ General Practice Alliance, Shenzhen, Guangdong, China; ^3^ University Town East Community Health Service Center, Shenzhen, Guangdong, China; ^4^ Department of Otorhinolaryngology Head and Neck Surgery, Shenzhen Children’s Hospital, Shenzhen, Guangdong, China; ^5^ Ophthalmology Department, Shenzhen OCT Hospital, Shenzhen, Guangdong, China

**Keywords:** computer-aided diagnosis, fundus camera, ophthalmology, image classification, Inception V3, Resnet-50

## Abstract

**Purpose:** We aim to present effective and computer aided diagnostics in the field of ophthalmology and improve eye health. This study aims to create an automated deep learning based system for categorizing fundus images into three classes: normal, macular degeneration and tessellated fundus for the timely recognition and treatment of diabetic retinopathy and other diseases.

**Methods:** A total of 1,032 fundus images were collected from 516 patients using fundus camera from Health Management Center, Shenzhen University General Hospital Shenzhen University, Shenzhen 518055, Guangdong, China. Then, Inception V3 and ResNet-50 deep learning models are used to classify fundus images into three classes, Normal, Macular degeneration and tessellated fundus for the timely recognition and treatment of fundus diseases.

**Results:** The experimental results show that the effect of model recognition is the best when the Adam is used as optimizer method, the number of iterations is 150, and 0.00 as the learning rate. According to our proposed approach we, achieved the highest accuracy of 93.81% and 91.76% by using ResNet-50 and Inception V3 after fine-tuned and adjusted hyper parameters according to our classification problem.

**Conclusion:** Our research provides a reference to the clinical diagnosis or screening for diabetic retinopathy and other eye diseases. Our suggested computer aided diagnostics framework will prevent incorrect diagnoses caused by the low image quality and individual experience, and other factors. In future implementations, the ophthalmologists can implement more advanced learning algorithms to improve the accuracy of diagnosis.

## Introduction

**FIGURE 1:**
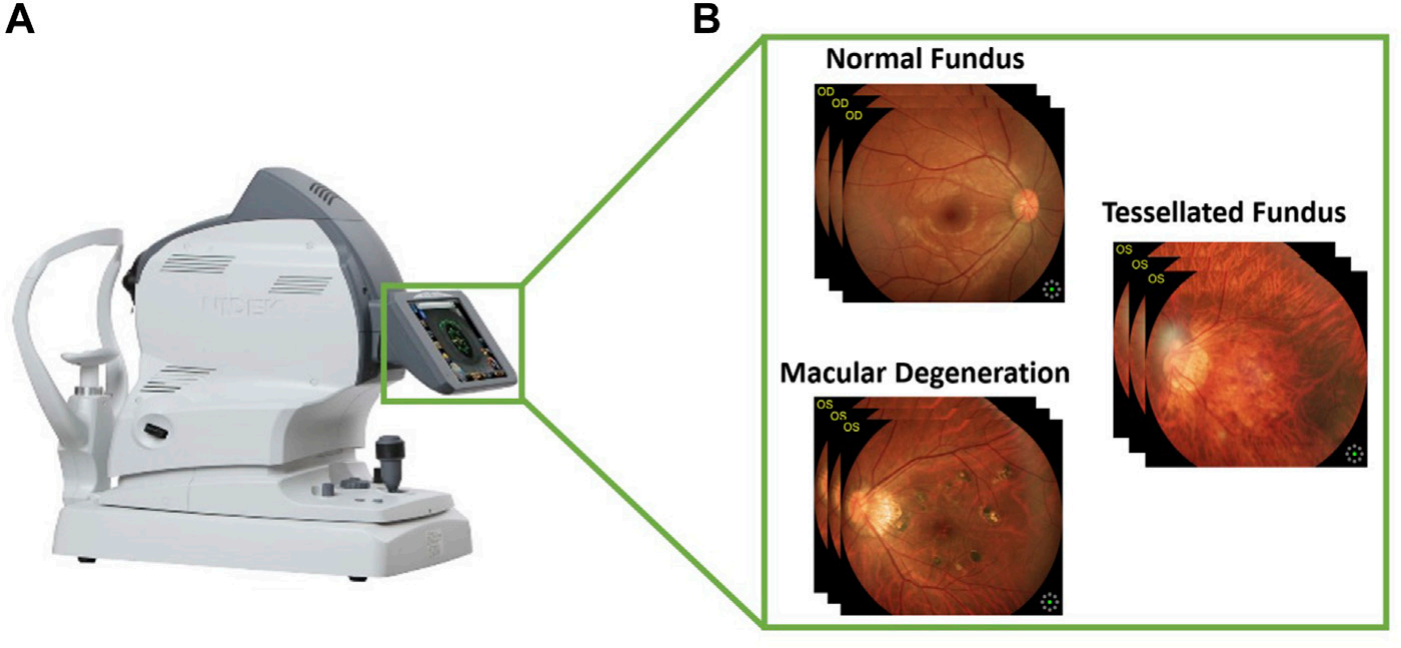
Visual representation of collecting images from fundus camera. **(A)** Fundus camera and **(B)** the fundus images according to three classes.

The fundus is the posterior part of the eye, which includes the retina, choroid, photoreceptor cells, as well as the blood vessels and the optic nerve. Fundus diseases can affect any of these structures, leading to a range of symptoms including vision loss, blind spots, and difficulty seeing in low light. These diseases can be caused by a variety of factors, including genetics, aging, and chronic health conditions like diabetes. Macular degeneration is a medical condition that affects the macula, which is the part of the eye responsible for central vision and this condition can cause a loss of central vision. Some of the known risk factors for this condition include age, smoking, obesity, and a history of high blood pressure. On the other hand, a tessellated fundus is a term used to describe the appearance of the retina at the back of the eye when viewed through an ophthalmoscope. It is characterized by a mosaic-like pattern of light and dark areas, which is caused by the different layers and structures of the retina being visible through the ophthalmoscope. When viewed through an ophthalmoscope, the different layers of the retina, including the photoreceptor cells, the blood vessels, and the nerve fibers, can all be seen, creating the characteristic tessellated pattern. Figure 1 illustrates the process of collecting data from fundus camera.

Fundus images are typically taken using specialized equipment, such as an ophthalmoscope or a fundus camera. These images can provide important information about the health of the eye and can be used by doctors to diagnose and monitor eye diseases. Healthcare provider will review the images carefully, looking for any abnormalities or signs of disease. They may also compare the images to a reference image or to images taken at an earlier time, to track any changes in the condition of the eye. This means that not all healthcare providers have the necessary training and experience to accurately diagnose eye diseases using fundus images. In addition, fundus images can be affected by factors such as the position of the eye, the lighting conditions, and the quality of the imaging equipment, which can affect their accuracy and reliability. Even trained ophthalmologists do not grade retinal images consistently, with significant variability in sensitivity for detecting retinal diseases (Burlina et al., 2017a). These limitations highlight the importance of computer-aided framework to classify fundus images into different disease diagnosed. Deep learning technology has recently used in various application of life including image classification (Bukhari et al., 2022), text analytics (Hussain et al., 2022; Wahid et al., 2022), crisis management (Sahar et al., 2022) and in medical imaging (Wong et al., 2021).

Deep learning algorithms can automate expert-level diagnostic tasks in ophthalmology, such as the diagnosis of glaucoma (Liu et al., 2018), age-related macular degeneration (Burlina et al., 2017b), and diabetic retinopathy (Gargeya and Leng, 2017; Ting et al., 2017) utilizing retinal fundus pictures. The authors of (Tan et al., 2017) used a single framework to autonomously segment exudates, micro aneurysms, and hemorrhages using a 10 layer convolutional neural network (CNN). Combining handcrafted (Annunziata and Trucco, 2016) data taken from the green channel of the normalized and equalized image, deep learning feature vectors were built using four convolutional layers and one fully connected layer from CNN (trained using LeNet architecture). Using EyePACS, the authors of (Lam et al., 2018) were able to identify the presence of five different kinds of red lesions, including normal, micro aneurysms, hemorrhages, exudates, and retinal neovascularization.

The authors of (Liskowski and Krawiec, 2016) proposed a deep learning based blood vessel segmentation framework of retinal fundus images datasets around the same time that (Maji et al., 2015) used a hybrid of random forest and deep neural network (DNN) for blood vessel segmentation. Their experimental results outperformed many existing approaches. In order to predict the severity of age-related macular degeneration, the study (Taibouni et al., 2021) suggested a classification architecture based on deep learning (AMD). An ensemble of various convolutional neural networks were employed in this study to classify AMD into 13 different categories (Ying et al., 2009). The paper (Govindaiah et al., 2018) described an expanded examination of (Burlina et al., 2017a) using a deeper VGG-16 architectural modification. Images were scaled to a standard reference level and the macula was selected as a Region of Interest. For comparison with the VGG-16, a 50 layer Keras implementation of residual neural network was used.

## Materials and methods

**FIGURE 2:**
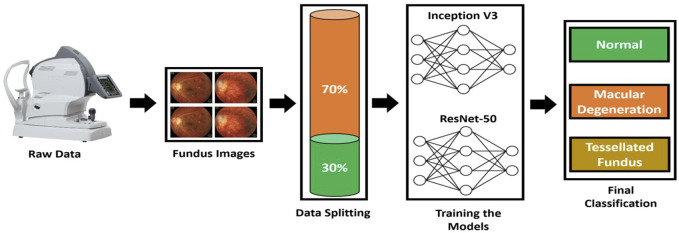
Proposed Framework to classify fundus images. A domain specific image is collected from fundus camera; then, data is split to train and test set. Inception V3 and VGG-16 are trained according to the dataset to classify fundus images.

To increase and strengthen the diagnostic capability, a computer-aided autonomous framework is required to divide Fundus images into three categories. Recently, deep learning technology has dominated contemporary science and technology, permeating a number of medical research fields (Gargeya and Leng, 2017). Deep learning technology has the potential to completely utilize enormous amounts of data, automatically understand the features in the data, support physicians in diagnosis accurately and quickly, and improve medical efficiency. For the purpose of early detection and treatment, our research applied a deep learning framework based on transfer learning to fundus images and divided them into three classes: normal, macular degeneration, and tessellated fundus. We used pre-trained models from ResNet-50 and Inception V3, tweaked them, and changed hyper parameters in accordance with our classification issue. The proposed framework to address the mentioned research gap is shown in Figure 2.

### Data collection and statistics

**FIGURE 3:**
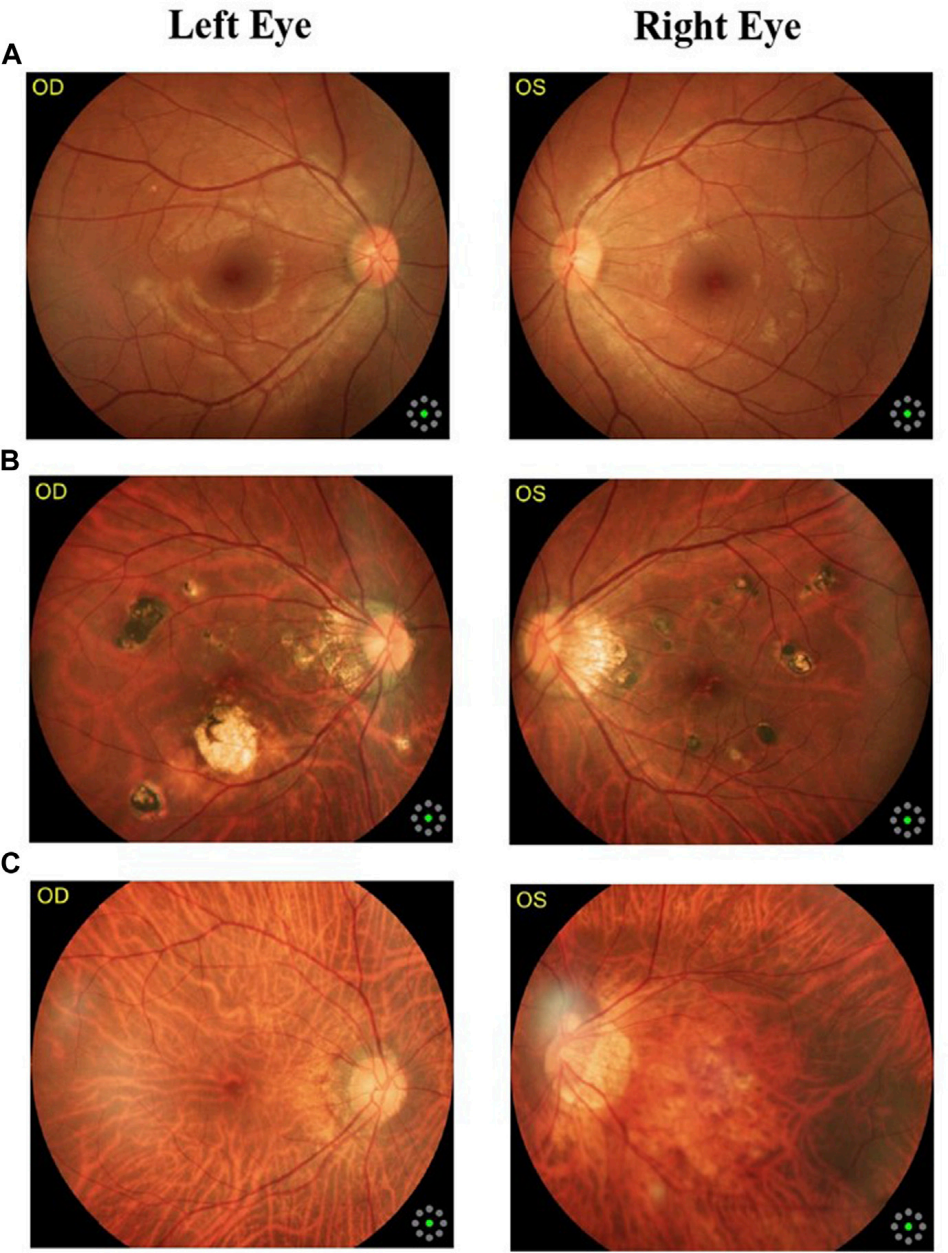
Samples of fundus images in various stages. **(A)** normal, **(B)** macular degeneration and **(C)** tessellated fundus.

Fundus cameras are specialized medical devices that are used to take images of the retina, which is the light-sensitive tissue at the back of the eye. These cameras use a combination of lenses and light sources to capture detailed images of the retina (Gargeya and Leng, 2017). The resulting image will be a detailed, high-resolution of the retina, which the doctor can then use to diagnose and treat any eye conditions that the patient may have. In this study, a total of 1,032 fundus images were collected from 516 patients using fundus camera from Health Management Center, Shenzhen University General Hospital Shenzhen University, Shenzhen 518055, Guangdong, China. The sample dataset is shown in Figure 3.

Moreover, we also described the statistics of our dataset in the table form collected from 516 patients as shown in Table 1. Finally we split our dataset in the training and testing set with ratio of 70% and 30%, respectively.

**TABLE 1:** Statistics of our dataset in each class.

	Normal	Macular degeneration	Tessellated fundus
No. of Images	364	329	339

### Inception V3

Inception-v3 is a 48-layer deep pre-trained convolutional neural network model, as shown in Eq. 1 and it is able to learn and recognize complex patterns and features in medical images. One of the key features of Inception V3 is its ability to scale to large datasets and to handle images of varying sizes and resolutions. This is important in the field of medical imaging, where images can vary greatly in terms of size, resolution, and quality. Typically, the Inception module includes one maximum pooling and three convolutions of various sizes (Ting et al., 2017). After the convolution operation, the channel is aggregated for the network output of the preceding layer, and the non-linear fusion is then carried out. In this model, over-fitting can be avoided while enhancing the network’s expression and flexibility to various scales.

$$A_{X}=\left[\begin{array}{ccc} A_{1,1} & \ldots & A_{1N}\\ A_{21} & \ldots & A_{2N}\\ A_{M1} & \ldots & A_{MN} \end{array}\right]*\left[\begin{array}{ccc} B_{1,1} & \ldots & B_{1N}\\ B_{21} & \ldots & B_{2N}\\ B_{M1} & \ldots & B_{MN} \end{array}\right]=\overset{M-1}{\underset{i=0}{\sum }}\overset{N-1}{\underset{j=0}{\sum }}A_{\left(M-1\right),\left(N-j\right)}B_{\left(i+1\right),\left(j+1\right)}$$ (1)

To avoid overfitting, we flattened the output layer and reduced its dimensions to one. We then added a sigmoid layer for classification, as well as a fully connected layer with 1,024 hidden units, a Relu activation function as shown in Eq. 2, and a dropout with the rate of 0.4. The weights of the classification layers were initialized using the algorithm described in (Beauxis-Aussalet and Hardman, 2014) as shown in Eq. 3. This approach allowed us to effectively use Inception V3 for our purposes.

$$f\left(x\right)=\max\left(0,x\right)$$ (2)

$$W_{k}\;∼\;U\left(-\frac{1}{\sqrt{m}},1/\sqrt{m}\right)$$ (3)

Where e *U* (−*a*, *b*) is a uniform distribution in the interval [−*a*, *b*], m is the size of the previous layer, and *W*_*k*_ stands for weight parameters in the CNN at iteration *k*. The complete model architecture and hyper parameter details are shown in Table 2.

**TABLE 2:** Hyper Parameters details used in the Inception V3 model according to our dataset.

Layer (type)	Output shape	Param
inception_v3 (Model)	(None, 8, 8, 2048)	21,802,784
flatten_ 1 (Flatten)	(None, 131072)	0
activation_95 (Activation)	(None, 131072)	0
dropout_ 1 (Dropout)	(None, 131072)	0
dense_ 1 (Dense)	(None, 1,024)	1,34,218,752
activation_96 (Activation)	(None, 1,024)	0
dropout_2 (Dropout)	(None, 1,024)	0
dense_2 (Dense)	(None, 28)	28,700
activation_97 (Activation)	(None, 3)	0

### ResNet-50

**FIGURE 4:**
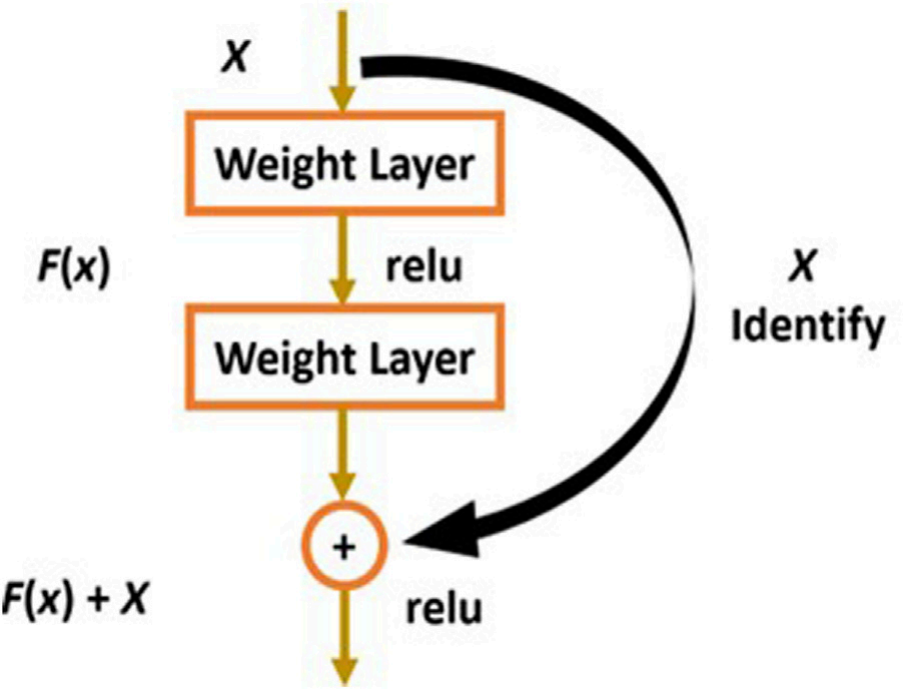
Residual blocks of ResNet Network.

ResNet-50 is a convolutional neural network with 50 layers that focuses on learning residuals as opposed to features (Litjens et al., 2017). This architecture introduces the idea of the Residual Network to address the issue of the vanishing/exploding gradient. We therefore learn a residual function H(x) rather than simply approximating the underlying mapping that we desire, H(x). As shown in Figure 4, this is accomplished by making the output of a stack of layers be y = F(x) + x, where F(x) is the output of the layers and the initial input x is added element by element.

Therefore, if our underlying mapping is still y = H(x) that we want to learn, then F(x) = H(x)−x so that y = F(x)+x = H(x)−x + x = H(x). The idea of learning identity mappings is now easier, since we just need to set all weights to 0, so that H(x) = 0 and F(x) = −x, so y = x is learned (Burlina et al., 2017b). Then comes the activation function, f() and we get the output as H(x) as shown in Eqs 4, 5 respectively.

$$H\left(x\right)=f\left(wx+b\right)$$ (4)

$$H\left(x\right)=f\left(x\right)+x$$ (5)

The complete model architecture and hyper parameter details are shown in Table 3, which consist of different layers including, convolutional, max pooling, flatten, dense, and dropout layer which mainly helpful to avoid the over-fitting of our model.

**TABLE 3:** Hyper Parameters details used in ResNet-50 model according to our dataset.

Layer (type)	Output shape	Param
conv2d (Conv2D)	(None, 26, 26, 28)	784
max_pooling2d	(None, 13, 13, 28)	0
conv2d_ 1 (Conv2D)	(None, 11, 11, 64)	16,192
max_pooling2d_ 1	(None, 5, 5, 64)	0
conv2d_2 (Conv2D)	(None, 3, 3, 64)	36,928
flatten (Flatten)	(None, 576)	0
dense (Dense)	(None, 640)	369,280
dropout (Dropout)	(None, 640)	0
dense_ 1 (Dense)	(None, 264)	169,224
dense_2 (Dense)	(None, 64)	16,960
dense_3 (Dense)	(None, 3)	260

All experiments in this paper are conducted on Intel(R) Celeron(R) CPU N3150 @ 1.60 GHz. The operating system is Windows 64-bit, Python 3.6.6, and TensorFlow deep Learning framework 1.8.0, and CUDA 10.1.

### Performance metrics

Evaluating the performance of a deep learning model is crucial in order to determine its ability to make accurate predictions on unseen data. By assessing the model’s performance, we can identify any potential limitations or issues and take steps to improve its accuracy. We assessed our model’s performance using accuracy and loss graphs as described in Eqs 6, 7 respectively.

$$Accuracy=\frac{TP+TN}{TP+TN+FP+FN}$$ (6)

The total number of correctly detected positive instances is known as true positive (TP). True negative (TN), false positive (FP), and false negative (FN) are the percentages of false positive and false negative occurrences with ground truth, respectively, that are correctly classified as positive and negative cases (cases without stenosis) (Davis et al., 2009).

$$L\left(W\right)=-\frac{1}{n}\;\overset{N}{\underset{n=1}{\sum }}\left(y_{n}\log\left(y_{n}\right)+\left(1-y_{n}\right)\log\left(1-y_{n}\right)\right)$$ (7)

Where y is the input patch’s ground truth label, calculating the gradient of the function L for the network weights W minimizes the loss function during the model training process.

In addition, we assessed the performance of our model using a confusion matrix. The columns of the matrix indicate forecasts of class instances, while the rows of the matrix show actual class instances. The fact that this matrix can determine whether the machine has mixed up two classes is whence its name originates (Liu et al., 2014). Following is a list of rates for a binary classifier that are frequently determined using a confusion matrix (Guan et al., 2019):

Recall is a measure of a model’s ability to correctly identify all relevant instances from a dataset as shown in Eq. 8.

$$Recall=\frac{TP}{TP+FN}$$ (8)

In Eq. 9, the mathematical expression for precision is displayed, and it is indicated that it is a measure of a model’s ability to correctly identify only relevant instances from a dataset.

$$Precision=\frac{TP}{TP+FP}$$ (9)

### Experimental results

**FIGURE 5:**
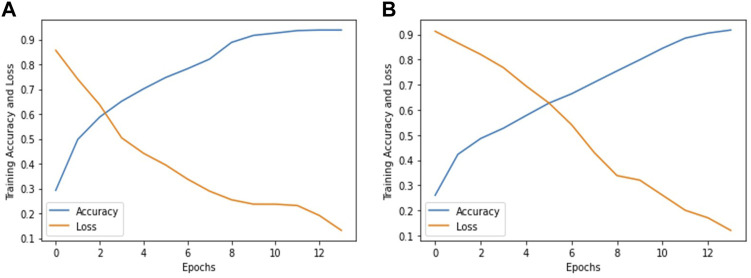
Accuracy and loss graph according to our dataset. **(A)** represents the accuracy and loss graph using ResNet-50 and **(B)** represents the accuracy and loss graph using Inception V3.

In this research work, we used pre-trained deep learning models to improve the diagnosis and early treatment of various fundus diseases. The Inception V3 and ResNet-50 deep learning models are used to classify fundus images into three classes, Normal, Macular degeneration and tessellated fundus for the timely recognition and treatment of fundus diseases. After fine-tuning and adjusting hyper parameters in accordance with our classification problem, we used ResNet-50 and Inception V3 to reach the greatest accuracy of 93.81% and 91.76%, respectively, utilizing our proposed method. Figure 5 accuracy and loss graph was used to evaluate how well our models performed. In Figures 5A, B show the accuracy and loss graphs for classifying fundus images into three categories: normal, macular degeneration, and tessellated fundus using ResNet-50 and Inception V3, respectively.

**FIGURE 6:**
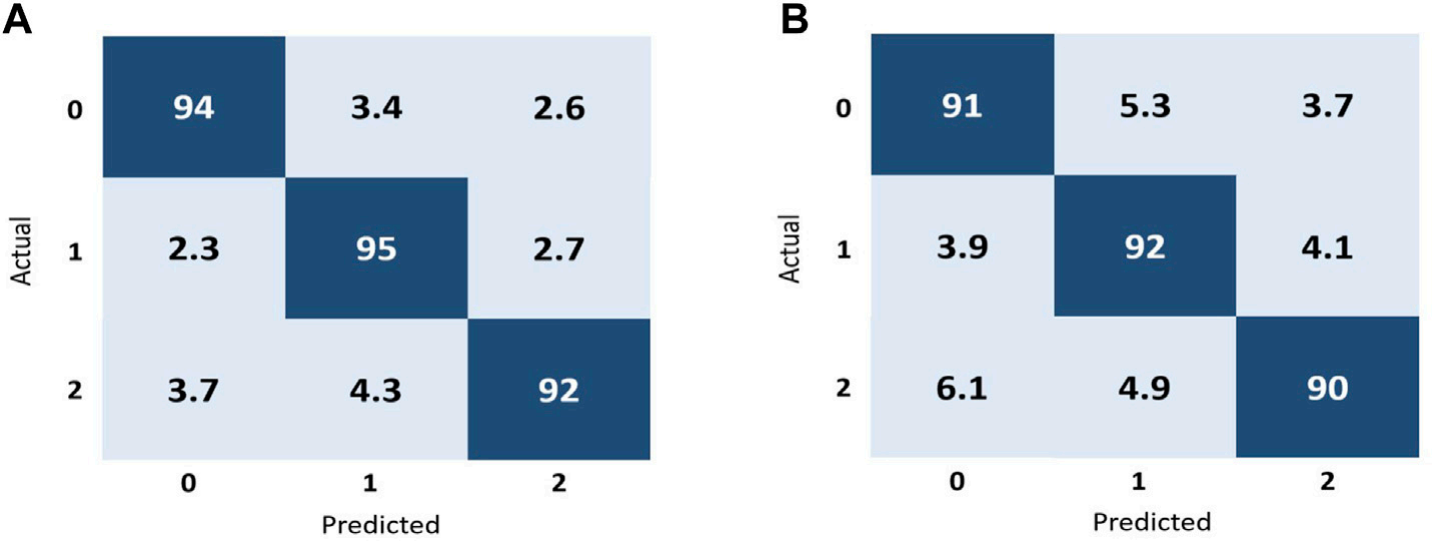
Performance representation of our trained models using confusion matrix. **(A)** represents the performance of ResNet-50 and **(B)** represents the performance of Inception V3 according to our dataset.

In the context of image classification, a confusion matrix can provide insight into which classes are being accurately predicted by the algorithm and which are not, as well as highlight any potential issues with the classifier. In the Figure 6A represents the confusion matrix using ResNet-50 and Figure 6B represents the confusion matrix using Inception V3 to classify fundus images into three classes: normal, macular degeneration and tessellated fundus.

As shown in Figure 6A, for the class 0, 94% of the data correctly classified in this class known as true positive (TP), in that case in false negative (FN) is equal to 6, while the false positive(FP) for that class is also six and the value for true negative for the class 0 is 194. Moreover for the better interpretation of the performance of our proposed models and evaluation metric for each individual fundus disease, we calculated accuracy, precision, recall and f-measure for each individual classes by using both models. We randomly selected one image from each class and perform validation on both models, the validation results are shown in Tables 4, 5. Tables 4, 5 represent the performance metrics for each individual classes of fundus diseases using ResNet-50 and Inception V3, respectively.

**TABLE 4:** Performance evaluation for each class using.

Class	Accuracy	Precision	Recall	F1 score
Class 0 (Normal) 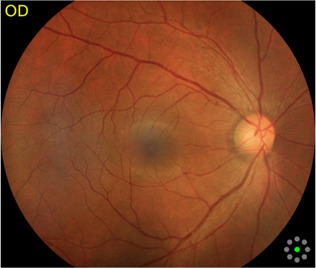	0.9567	0.94	0.93	0.94
Class 1 (macular degeneration) 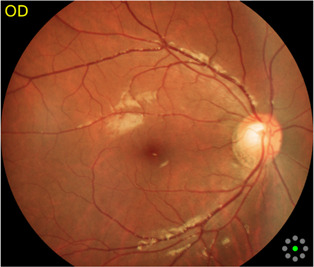	0.9567	0.95	0.92	0.94
Class 2 (tessellated fundus) 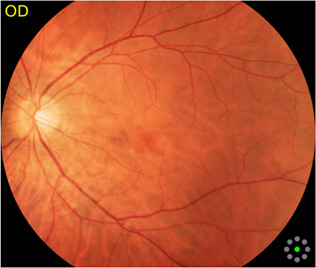	0.96	0.92	0.96	0.94

**TABLE 5:** Performance evaluation for each class using.

Class	Accuracy	Precision	Recall	F1 score
Class 0 (Normal) 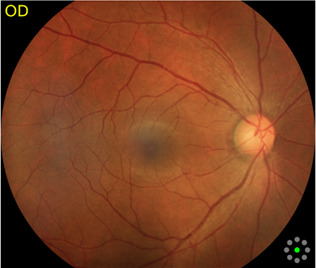	0.94	0.91	0.91	0.91
Class 1 (macular degeneration) 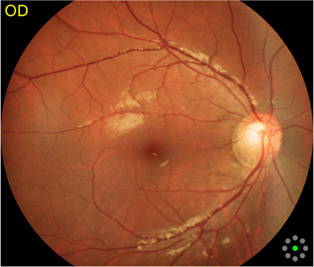	0.9433	0.92	0.91	0.92
Class 2 (tessellated fundus) 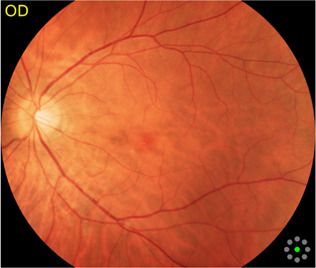	0.9367	0.9	0.91	0.9

## Discussion

The fundus is the posterior part of the eye, which includes the retina, choroid, photoreceptor cells, as well as the blood vessels and the optic nerve. Fundus diseases can be caused by a variety of factors, including genetics, aging, and chronic health conditions like diabetes. Macular degeneration is a medical condition that affects the macula, which is the part of the eye responsible for central vision and this condition can cause a loss of central vision. A tessellated fundus is a term used to describe the appearance of the retina at the back of the eye when viewed through an ophthalmoscope. The most common cause of visual impairment is cataract, which caused 55.0% blindness and 50.2% low vision. Fundus lesions were second, causing 22.9% (blindness) and 23.8% (low vision), whereas glaucoma was rare, causing only 9.6% (blindness) and 1.7% (low vision) (Liu et al., 2018). . In addition, fundus images can be affected by factors such as the position of the eye, the lighting conditions, and the quality of the imaging equipment, which can affect their accuracy and reliability. Even skilled ophthalmologists do not consistently evaluate retinal images, and the sensitivity for identifying retinal disorders varies widely (Burlina et al., 2017a). These drawbacks emphasize the value of using a computer-aided framework to categorize fundus images according to various diseases. A total of 1,032 fundus photos from 516 patients were gathered for this study utilizing a fundus camera at the Health Management Center of Shenzhen University General Hospital in Shenzhen, Guangdong, China. We fine-tuned and adjust the hyper parameters of Inception V3 and ResNet-50 according to our dataset as shown in Tables 4, 5 respectively. According to our proposed approach we, achieved the highest accuracy of 93.81% and 91.76% by using ResNet-50 and Inception V3 as shown in Figure 5. Finally to perform the validation of our models, we randomly selected one image from each class and perform validation on both models, the validation results are shown in Tables 4, 5. Tables 4, 5 represent the performance metrics for each individual classes of fundus diseases using ResNet-50 and Inception V3, respectively. Moreover, we also compared the performance of our proposed models with prior studies in this domain as shown in Table 6.

**TABLE 6:** Comparative accuracy of our proposed model with previous studies.

Studies	Techniques	Accuracy (%)
Lachure et al. (2015)	GLCM + SVM	82
Priya and Aruna (2012)	SVM + NN	86.9
Abràmoff et al. (2010)	FCM, NN, shape	93
**Proposed approach**	**Pre-trained DL Models**	**93.81**

The purpose of bold is to highlight the resultachieved by this study, which is significant.

Our suggested framework for computer-aided diagnosis will stop inaccurate diagnoses brought on by poor image quality, individual differences in expertise, and other reasons. To increase the accuracy of the diagnosis, ophthalmologists can use more sophisticated learning algorithms in the future.

## Limitation and future work

Deep learning requires a large amount of data to improve performance and avoid over-fitting. It is difficult to acquire medical imaging data of low-incidence serious diseases in general practice. Due to differences in patients and the appearance of the prostate, future work will focus on testing the model with a more extensive data set. In future implementations, the ophthalmologists can implement more advanced learning algorithms to improve the accuracy of diagnosis. In the future, we could be able to better localize the lesions by developing a unique object detection model and enhancing the CNN512’s performance classification by including more layers. The system may perform better after being tested and tuned with datasets that are more evenly distributed.

## Conclusion

This study concluded that ResNet-50 and Inception V3 methods for fundus disease classification into three classes are the most promising and successful. The first class normal fundus image, second is Macular degeneration and third is tessellated fundus. According to our proposed approach we, achieved the highest accuracy of 93.81% and 91.76% by using ResNet-50 and Inception V3, respectively. Our research provides a reference to the clinical diagnosis or screening for diabetic retinopathy and other eye diseases. Our suggested computer aided diagnostics framework will prevent incorrect diagnoses caused by the low image quality and individual experience, and other factors.

## Data Availability

The original contributions presented in the study are included in the article/Supplementary Material, further inquiries can be directed to the corresponding authors.
